# Academic grit scale for Chinese middle- and upper-grade primary school students: testing its factor structure and measurement invariance

**DOI:** 10.1186/s40359-024-01622-y

**Published:** 2024-03-14

**Authors:** Rongmao Lin, Yanping Chen, Yilin Shen, Ting Hu, Ying Huang, Yishan Yang, Xueting Yu, Jinliang Ding

**Affiliations:** 1 School of psychology, Fujian Normal University, Fuzhou, 350117 China; 2 Department of Social Psychology, University of Groningen, Groningen, 9712 TS The Netherlands; 3 School of Humanities and Teacher Education, Wuyi University, Wuyishan, 354300 China

**Keywords:** Academic grit scale, Academic grit, Factor structure, Measurement invariance, Middle- and upper-grade primary school students

## Abstract

The Academic Grit Scale (AGS) is a novel measure of academic-specific grit. However, its factor structure and measurement invariance have yet to be thoroughly supported. The present study tested the factor structure and measurement invariance of the AGS with a large sample of early adolescents (aged 9–14 years) from China (*N* = 1,894). The bifactor model showed that the AGS was predominately accounted for by the general factor rather than the domain-specific factors; the parallel model from the AGS’s one-factor model showed good fit indices; thus, the AGS should be described as a univocal solution and reported as the total score. Gender and grade measurement invariance were supported at a scalar level, warranting further mean difference comparisons. In addition, academic grit was significantly associated with positive academic emotions and academic achievement, yielding evidence of good criteria-related validity. The current study contributes additional evidence to the construct validity of the Chinese version of the AGS among middle- and upper-grade primary school students in China.

**Supplementary Information:**

The online version contains supplementary material available at 10.1186/s40359-024-01622-y.

## Introduction

Academic grit commonly refers to the determination, resilience, and focus that students demonstrate in their pursuit of academic excellence [1–3]. It is an important characteristic or skill for individuals to maximize their academic potential and achieve their academic goals [1]. During the early stage of adolescence, individuals experience a pivotal period characterized by significant cognitive and emotional development [4]. Relatedly, this phase also marks the start of heightened scholastic expectations and the introduction of more intricate coursework [5]. The development of academic grit at this phase is highly significant, as it may establish the groundwork for developing individuals’ abilities for learning and emotional adjustment, improving their academic performance, and facilitating their long-term success [2, 6, 7]. In particular, in the context of China, high academic pressure and intense academic competition have prompted a pressing need for a more comprehensive understanding of Chinese early adolescents’ academic grit [8, 9]. Before comprehending academic grit, it is necessary to possess a reliable and valid evaluation tool for evaluating academic grit. Unfortunately, the measurement instrument for academic grit remains limited. The Academic Grit Scale (AGS) [1], the only commonly accepted measure of academic grit, lacks predictions regarding its construct validity (i.e., factor structure and measurement invariance) among Chinese middle- and upper-grade primary school students. In order to assure the effective application of the AGS instrument among Chinese early adolescents, this study aims to examine AGS’s construct validity, specifically focusing on its factor structure and measurement invariance, within the population of Chinese early adolescents in middle- and upper-grade primary schools^1^.

### Outline of the AGS

Academic grit is a subdomain of grit. Grit is described as a passion for long-term goals and a willingness to persevere, determining if individuals successfully maximize their potential in a variety of domains [2]. In contrast, academic grit emphasizes individuals’ grit specifically in their academic lives. As stated by Duckworth and Quinn [3], individuals may exhibit considerable grit in their professional lives (for example, in the academic domain) but very little in their daily struggles. To specifically measure an individual’s grit in the academic domain, Clark and Malecki [1] developed the Academic Grit Scale (AGS) to assess a youth’s commitment to their long-term academic goals. The AGS is a 10-item, one-factor construct with three aspects: determination, resilience, and focus. It was found to have good content and face validity among American middle school students. Besides, a reliability test showed evidence of high internal consistency and reliability of AGS (Cronbach’s *α* = 0.94), and a confirmatory factor analysis revealed good construct validity of AGS. Furthermore, AGS not only represented good criterion-related validity in association with academic achievement, life satisfaction, and school satisfaction but also exhibited better predictive validity on these school outcomes than general grit [1].

### Factor structure of the AGS

The AGS’s factorial structure should be further tested. Firstly, the extant evidence may not adequately support the univocal model of AGS. In Clark and Malecki’s [1] research, it has been shown that a key homogeneous factor accounts for 50.32% of AGS’s total variance, but little is known about AGS’s heterogeneity (i.e., the degree of differences between AGS’s three components, determination, resilience, and focus). They revealed a one-factor model under exploratory factor analysis (EFA) and only tested a global model with confirmatory factor analysis (CFA). However, their theoretical model (i.e., the three-factor model) has not yet been validated under CFA to further identify their priori theory, and there is no sufficient evidence to show that AGS’s heterogeneity can be disregarded. In other words, it’s unclear whether AGS is just a global construct or if it is a single, cohesive construct with three subscales. Second, there was not a full psychometric evaluation of how to use the AGS, such as using it as a total score or three factor scores. In many cases, researchers often use a scale as a total score or directly apply the results from validated models to scoring methods [10]. Although logically sound, this approach is too inaccurate to know about its tenability. In particular, McNeish and Wolf [10] note that when evidence of validity from factor analysis is applied to the sum scores, there may be unexpected bias, and the score interpretation of the solution found through validated models could be wrong. Indeed, they recommend using a parallel model because the parallel model is a perfect linear transformation of sum scores and factor scores.

To solve the above issues, we use both CFA and a bifactor model approach to test the AGS’s factor structure first. After that, we fit the one-factor and three-factor parallel models^2^ of AGS separately to identify how to score AGS. First, the CFA and the bifactor model approach can help identify the factor structure of AGS. Traditional CFA compares one-factor and three-factor models by using goodness-of-fit indices. The bifactor model embeds the one-factor model and the three-factor model into a framework and then estimates their variance simultaneously to know homogeneity and heterogeneity within AGS’s items directly by using several indices such as explained common variance (*ECV*) and percent uncontaminated correlations (*PUC*) [11, 12]. Based on homogeneity and heterogeneity within AGS’s items, we can better know to what extent AGS could be a one-factor model or a three-factor model. Additionally, the parallel model can help us detect how to scientifically use the AGS (i.e., ascertain the scoring method of the AGS). In the parallel model, all items/questions within the same factor are considered related to a targeted latent ability or a specific aspect of ability. Each item/question within the same factor contributes equally and holds the same importance. Thus, the parallel model assumes that all items/questions within the same factor equally reflect the targeted ability or a certain aspect of targeted ability, which is equivalent to the sum score or factor scores we commonly calculate. AGS’s one-factor and three-factor parallel models are ideal equivalent models of AGS’s sum scores and its factor scores, respectively [10]. By evaluating the adequacy of fit indices for AGS’s one-factor and three-factor parallel models, we might ascertain whether it is meaningful to calculate AGS’s sum scores and/or its factor scores.

### Measurement invariance of the AGS

Another thing to think about is whether or not the structure of the measure is the same for each subgroup, or, in other words, whether the measurement invariance (MI) is established so that students are assessed accurately. MI means each cluster member with the same level of the trait has an equal probability of obtaining the same score on the test [13, 14], which is a prerequisite for group mean comparisons [15, 16]. Early adolescents are at a key point in physical and psychological growth [4]. A lot of changes happen in their daily lives. In particular, research has shown that early adolescents’ cognitive capacities vary between genders and grade levels [17–19], which may lead to differing perceptions and experiences of academic grit across genders and grades. In order to avoid potential biases related to gender and grade, it is imperative to establish MI before conducting comparisons of mean differences in academic grit across various gender and grade subgroups.

### Criteria-related validity of the AGS

We also took criteria-related validity into account. Positive academic emotion and academic performance play an important role in students’ academic lives and could be suitable criteria for examining the AGS. First, a wealth of empirical studies has yielded evidence of a positive link between grit and academic achievement in youth populations [see 20, for a review]. Research on domain-specific grit has also indicated that individuals with high levels of academic grit tend to have higher GPAs [1]. On the other hand, grit has been found to have a beneficial impact on individuals’ positive emotions and subjective well-being [21–23]. These evidences, including grit in relation to academic achievement and positive emotions, provide some insights into the relationship between academic grit and positive achievement and academic emotions. In other words, positive achievement and academic emotions could be good criteria for examining AGS. In our research, three criteria related to academic emotions and achievement—positive high arousal of academic emotions, positive low arousal of academic emotions, and academic achievement—were used to test the criteria-related validity of the AGS.

## The current research

Even though academic grit is important in psychological research and educational practice, the psychometric properties of AGS, a commonly accepted measurement tool, lack full inspection, especially among Chinese early adolescents. The main goal of this research was to inspect the AGS’s factor structure and measurement invariance among Chinese middle- and upper-grade primary school students. Due to the sparse evidence of the AGS in early adolescents, its factor structure and MI were exploratory.

## Materials and methods

### Participants

A sample group of 1,916 primary school students was recruited from four primary schools—two in Fujian Province and two in Jiangxi Province, People’s Republic of China. We employed a convenience sampling method to select these schools. In each school, stratified cluster sampling was conducted. Specifically, we considered grades as layers and recruited students from 3rd, 4th, 5th, and 6th grades. We then treated each class as a cluster and selected four classes from each grade. Therefore, a total of 64 classes were recruited. Excluding invalidated responses (i.e., unfilled contents with more than 5 items or all responses repeating the same option), 1,894 participants were retained (the retained rate was 98.85%). Of this total, 935 were boys (49.40%) and 959 were girls (50.60%); the numbers of participants in the 3rd, 4th, 5th, and 6th grades were 371 (19.60%), 557 (29.40%), 451 (23.80%), and 515 (27.20%), respectively. The mean age of the participants was 11.1 years (*SD* = 1.1), and their ages ranged from 9 to 14 years. Informed consent was obtained from the participants’ head teachers and parents. The Ethics Committee of the School of Psychology of Fujian Normal University in the People’s Republic of China reviewed and approved all procedures in this study.

### Measures

#### Academic grit scale, AGS

The AGS, a 10-item self-reported measure, was first made by Clark and Malecki [1]. It is used to evaluate the levels of adolescents’ academic grit. AGS is a one-factor structure with 4 items about determination, 4 items about resilience, and 2 items about focus. Items were measured on a 5-point Likert scale ranging from 1 (*not at all like me*) to 5 (*very much like me*), with higher scores reflecting higher levels of academic grit. After obtaining approval from the authors, the AGS was translated into Chinese to assess Chinese early adolescents. According to well-established back-translation procedures for the cross-cultural study [24], two independent bilingual translators first translated the English version into Chinese, and then another two bilingual translators performed a blind-back translation. If there was no discernible difference compared with the initial scale, the item in Chinese was kept; otherwise, the items were retranslated by a fifth translator. This process went on until all the items were retained in the Chinese version. Finally, a committee comprised of all the translators also reviewed the translations and backtranslations and produced a final version (see Appendix for the Chinese version of the Academic Grit Scale).

Besides, three psychological experts were invited to look at each item on the Chinese AGS scale for content validity and make suggestions about its relevance (i.e., whether the content of each item is enough to describe a youth’s academic grit) and semantic clarity (i.e., whether each item is clear and unambiguous). Based on the experts’ suggestions, the final version of the Chinese AGS scale was revised. Furthermore, 10 Chinese teachers in the primary school were asked to judge the face validity of each item (i.e., how fluent and clear the scale was), and the results showed that the revised version of the Chinese AGS scale had good face validity. Both Chinese and English versions of AGS can be seen in the appendix. In this study, the Cronbach’s α coefficient and the Omega coefficient (ω) for this scale were 0.88 and 0.94.

#### Academic emotion questionnaire, AEQ

The AEQ is used to measure students’ academic emotions [25]. Based on the degree of pleasure and arousal, AEQ is divided into 4 subscales: positive high-arousal emotions (i.e., pride, enjoyment, and hope), positive low-arousal emotions (i.e., contentment, calmness, and relief), negative high-arousal emotions (i.e., anxiety, shame, and anger), and negative low-arousal emotions (i.e., boredom, hopelessness, depression, fatigue, and sadness). Two subscales, positive high-arousal emotions and positive low-arousal emotions, were used in this study. Dong and Yu’s [25] research showed that Cronbach’s *α* coefficients for the two subscales were 0.79 and 0.82, respectively. Items were rated on a 5-point scale, ranging from 1 (*completely inconsistent*) to 5 (*consistent conformity*). The higher the score, the stronger the intensity of the corresponding academic emotional experiences [25]. In this study, Cronbach’s *α* coefficients for the two subscales were 0.88 and 0.92, and Omega coefficients (ω) for the two subscales were 0.87 and 0.92.

#### Academic achievement

Academic achievement was evaluated with three self-reported items: “On the last major exams (final, midterm, or monthly exam), my grade in Chinese/math/English was ____ (ranging from 0 to 100).” A mean score was calculated based on these standardized items to show a student’s academic achievement, with higher scores reflecting higher levels of academic achievement.

### Data analysis

All data processing was conducted in SPSS 24.0 and Mplus 8.0 for Windows [26, 27]. Preliminary data screening of distributions, skewness, and kurtosis was conducted in SPSS 24.0. CFA, multi-group structural equation modeling (MG-SEM), and a structural regression model were performed in Mplus 8.0. The item responses of the AGS generally exhibited normal distributions, but there were still slight deviations, with skewness values ranging from − 0.80 to -0.38 and kurtosis values ranging from − 0.65 to -0.23 [more than 0 but less than ± 1.96; see 28, 29]. We employed a robust maximum likelihood (MLR)^3^ estimation method for all AGS models to address minor deviations from normality in our data [30, 31].

#### Measurement models

Two hypothesized models, a one-factor model and a three-factor model, were tested with CFA. Model 1, originally supported by Clark and Malecki [1], was a unitary model in which all 10 items were loaded onto a single latent variable. Model 2 was a three-factor model with three correlated items: determination (items 1, 5, 9, and 10), resilience (items 2, 4, 6, and 8), and focus (items 3 and 7). This model was built on the theoretical concept of academic grit.

#### Evaluation of the bifactor model and the parallel models

Three domain-specific factors and a general factor (loaded by all 10 items) make up the AGS bifactor model. The domain-specific factors were specified as Model 2. Differing from Model 2, which specified the covariance of all factors in correlation with one another, Model 3 separately estimated the general and domain-specific factors and specified their variance as having no association [11].

When examining the construct of academic grit, three indices were computed: explained common variance (*ECV*), items for ECV (*I-ECV*), and percentage uncontaminated correlations (*PUC*). To be specific, *ECV* quantifies the extent to which a general factor can account for common variance, whereas *I-ECV* specifies the extent to which a general dimension can interpret each item’s variance. *PUC* demonstrates the percentage of AGS item correlations due to the general factor. According to the criteria of Rodriguez et al. [12], when *ECV* > 0.70 and/or *PUC* > 0.70, it is preferable to adopt a one-factor model; in cases where *ECV* and *PUC* are relatively small (*ECV* < 0.70 and *PUC* < 0.70), multidimensional models (i.e., the bifactor model and the three-factor model) should be further considered based on factor loadings.

When exploring the scoring methods of AGS, we fit the parallel models of one-factor and three-factor separately to further identify the tenability of sum scoring. The structures of the two parallel models are identical to our two hypothesized models (see the section on *Measurement Models*), but the error variance and loadings in the parallel models were constrained to be equal. Specifically, in the one-factor parallel model of the AGS, the error variance and loadings were set to be equal for 10 items. In the three-factor parallel model of the AGS, the loadings are set to 1 for all items; the error variances are distinct between factors but are constrained within factors.

#### Measurement invariance

Several fit indices were considered for model evaluation and comparison: the standardized root mean square residual (SRMR), the root mean square error of approximation (RMSEA), the comparative fit index (CFI), and the Tucker-Lewis index (TLI) [32]. Maydeu-Olivares [32] suggested that SRMR and RMSEA values should be less than or equal to 0.08, and lower values indicate better fit; CFI and TLI values should exceed or be equal to 0.90, and higher values mean a more ideal fit [32]. Besides, the Satorra-Bentler scaled chi-square (S-B *χ*^*2*^) is also considered for model evaluation and comparison. Similar to the chi-square (*χ*^*2*^), S-B *χ*^*2*^ is significantly influenced by sample size; however, it is considered to be a fundamental method of evaluation [31, 33, 34].

Moreover, configural (equal factor patterns), metric (same factor coefficients), and scalar invariance (equal indicator intercepts) tests were carried out sequentially. The latent mean difference test is meaningful if scalar invariance is reached in full or in part [35]. The changes in chi-squared value and CFI (*Δ*S-B *χ*^*2*^ and ΔCFI) were used when comparing the nested models. A significantly different S-B *χ*^*2*^ (*p* < .05) indicated the two adjacent models were significantly different [36]. Because the chi-squared difference test is vulnerable to sample size, the CFI change value (ΔCFI) was also employed in the model comparison. To indicate significant differences between the two models under consideration, the ΔCFI should be greater than 0.005 (ΔCFI > 0.005) [37]. If the differences were not significant, it means this kind of invariance is fully supported. If not, we will try to release constraints step by step to look for the possibility of partial invariance.

In addition, a structural regression model was also used to see if academic grit was correlated with positive high arousal of academic emotions, positive low arousal of academic emotions, and academic achievement.

## Results

### Confirmatory factor analysis

**Fig. 1:**
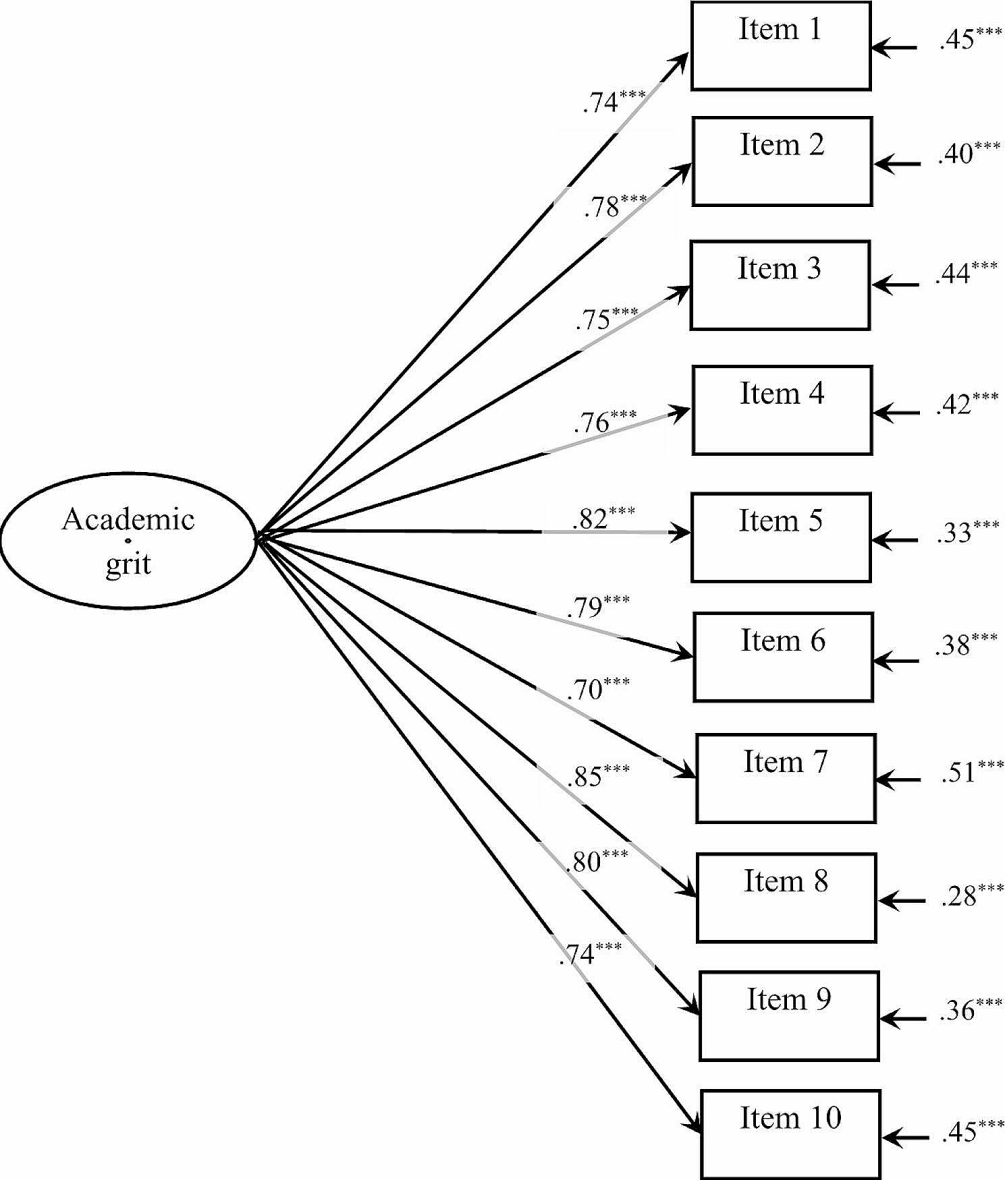
Factor structure and standardized loadings of one-factor model for the AGS. *Note*: ^***^p < .001

**Fig. 2:**
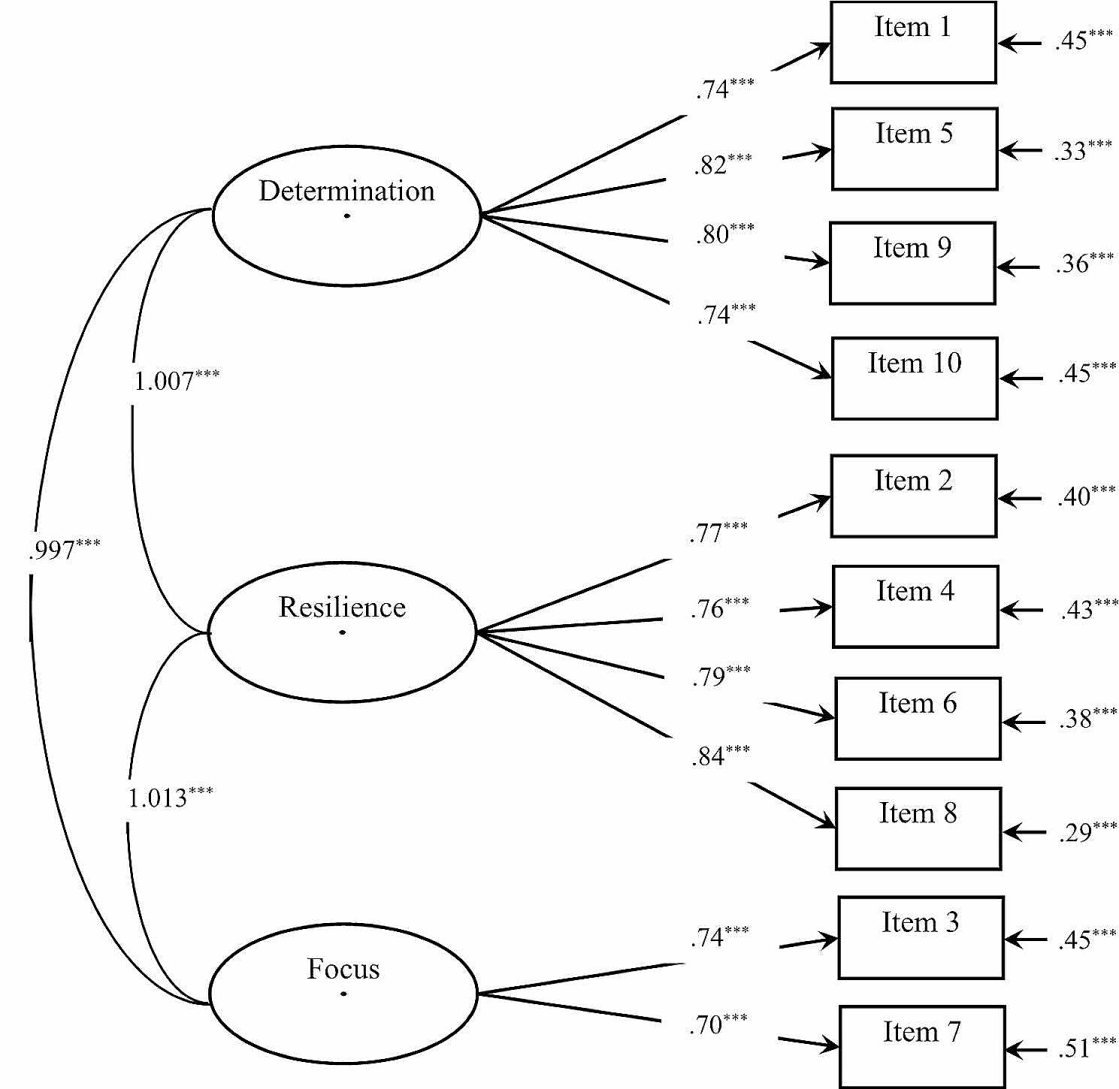
Factor structure and standardized loadings of three-factor model for the AGS. *Note*: ^***^p < .001

Table 1 presents the goodness-of-fit statistics for one-factor and three-factor models of the AGS. Figures 1 and 2 show the standardized coefficients for the two models. The values of both CFI and TLI for the two models were greater than 0.90; the RMSEA and SRMR were lower than 0.08. Both of the two models had good fit indices and did not differ meaningfully. Thus, they were simultaneously supported by the CFA^4^.

**Table 1:** Goodness-of-fit statistics for measurement models

Model	S-B χ^2^	df	CFI	TLI	RMSEA	SRMR
The one-factor model	157.56	35	0.980	0.974	0.043	0.021
The three-factor model	154.18	32	0.980	0.971	0.045	0.021

*Note*: Satorra-Bentler scaled ***χ***^***2***^: S-B ***χ***^***2***^

It is difficult to choose an optimal model by only considering goodness-of-fit. We then embedded both the one-factor and three-factor models into a larger model (called a bifactor model) to see which model described the AGS better.

### Evaluation of the bifactor model and the parallel models

**Fig. 3:**
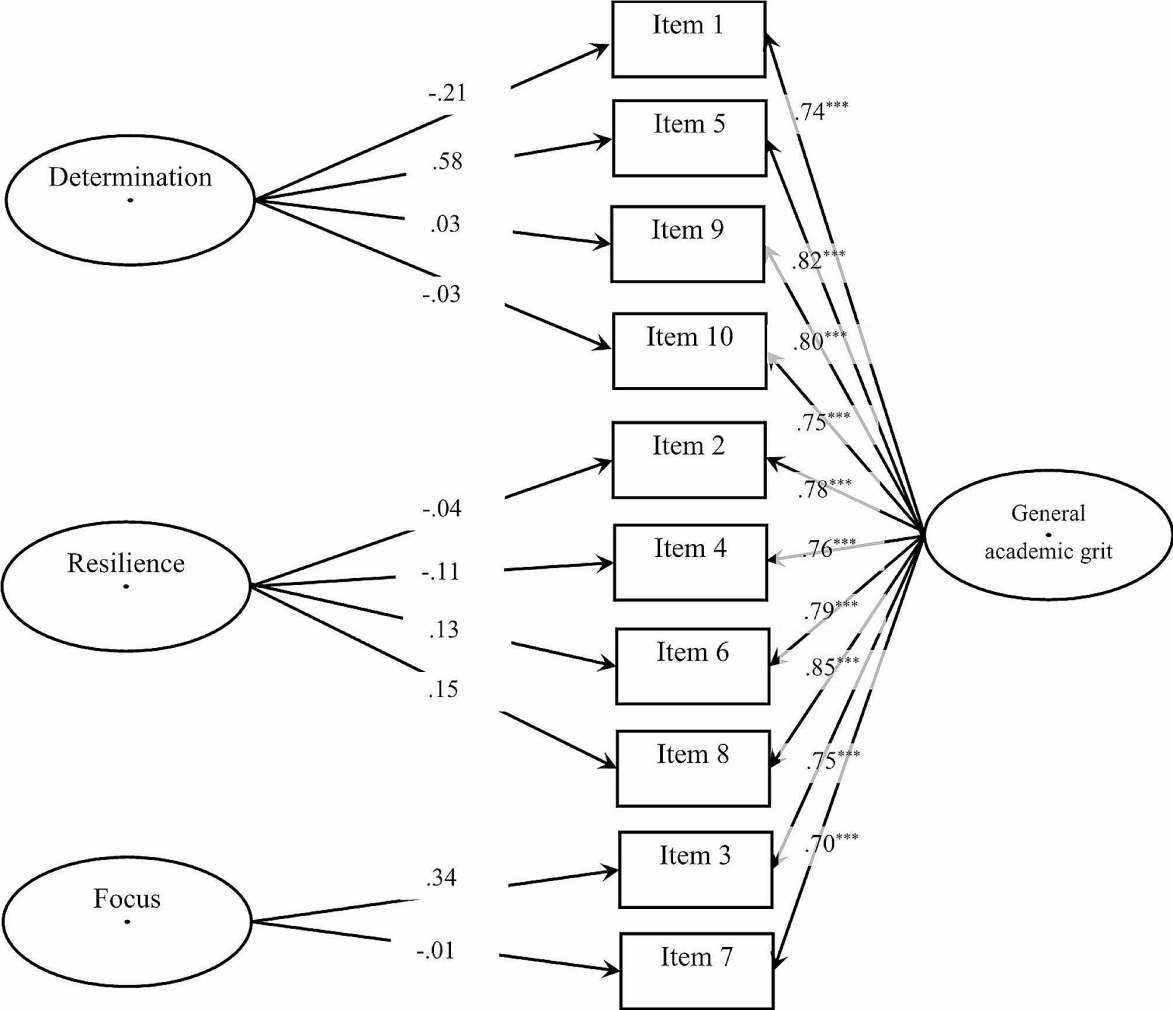
Factor structure and standardized loadings of bifactor model for the AGS. *Note*: ^***^: *p* < .001; considering the simplicity of the model, measurement errors for each item are not shown

Figure 3 presents the factor structure and standardized loadings of the bifactor model of the AGS. The bifactor model fit well: (S-B *χ*^*2*^ = 87.65, *df* = 53, CFI = 0.980, TLI = 0.964, SRMR = 0.021, RMSEA = 0.051). In the bifactor model, the *ECV* was 0.92, which meant that the general factor explained 92.00% of the common variance and that three group factors explained 8.00% of the remaining common variance. The average *I-ECV* value was 0.94 (from 0.67 to 1.00; see Table 2), implying that an average of 94.00% of the item common variance was interpretable by the general factor and the rest of 6.00% was attributable to domain-specific factors. The *PUC* was 0.71, indicating that the majority of the item correlations were concentrated in general academic grit.

**Table 2:** Standardized factor loadings for the AGS’s bifactor model

Item	General (SE)	F_1_ (SE)	F_2_ (SE)	F_3_ (SE)	I-ECV (SE)
1	0.74^***^(0.02)	− 0.02 (0.17)			1.00^***^(0.01)
5	0.82^***^(0.01)	0.58 (3.73)			0.67 (2.87)
9	0.80^***^(0.01)	0.03 (0.20)			1.00^***^(0.02)
10	0.75^***^(0.01)	− 0.03 (0.20)			1.00^***^(0.02)
2	0.78^***^(0.02)		− 0.04 (0.08)		1.00^***^(0.01)
4	0.76^***^(0.01)		− 0.11 (0.10)		0.98^***^(0.03)
6	0.79^***^(0.01)		0.13 (0.11)		0.97^***^(0.05)
8	0.70^***^(0.02)		0.15 (0.12)		0.97^***^(0.05)
3	0.75^***^(0.01)			0.34 (0.77)	0.83 (0.64)
7	0.70^***^(0.02)			− 0.01 (0.03)	1.00^***^(0.001)

*Note*: ^***^p < .001;

F_1_ = determination, F_2_ = resilience, F_3_ = focus; *I-ECV =* item explained common variance

Overall, high *ECV*, average *I-ECV*, and *PUC* values show that values show that there is a strong enough general factor to explain AGS’s internal structure, and the specificity of each factor (i.e., the percentage of variance that is uniquely explained by each factor) is small. Thus, a one-factor model better presents/explains the internal structure of AGS.

Furthermore, we fit a one-factor parallel model based on Fig. 1. The one-factor parallel model had good fit indices (S-B *χ*^*2*^ = 363.687, *df* = 53, CFI = 0.948, TLI = 0.956, SRMR = 0.056, RMSEA = 0.061), indicating that composing a total score in AGS is reasonable. We also fit a three-factor parallel model based on Fig. 2. The three-factor parallel model had poor model fit (S-B *χ*^*2*^ = 2686.286, *df* = 45, CFI = 0.560, TLI = 0.560, SRMR = 0.419, RMSEA = 0.176), indicating that computing the factor scores is not supported empirically.

Thus, good fit indices for the one-factor model and poor fit indices for the three-factor model show that it is reliable to use AGS as a total score, but it doesn’t make sense to figure out its factor scores.

### Measurement invariance

The AGS’s one-factor model, supported by the bifactor model, was used to look into its measurement invariance.

#### Measurement invariance across genders

The model fit indices for all measurement invariance tests are displayed in Table 3. The one-factor models for boys and girls fit well (see Models 1 and 2) and met the requirements of the MI. The model of configural invariance (Model 3) had an adequate fit, indicating that the structure of the AGS is similar for boys and girls. Subsequently, equal restrictions on all factor loading coefficients were tested across genders (Model 4). The results showed that, despite S-B *χ*^*2*^ significantly increasing (*p* = .018), the difference in CFI between Models 3 and 4 was small (0.001 < 0.005), suggesting that the metric invariance was reasonable across genders. Then, the indicator intercepts were constrained to be equal to test the scalar invariance (Model 5). In this case, S-B *χ*^*2*^ significantly increased (*p* = .01), but the change in CFI (0.002) was less than 0.005, showing that scalar invariance was supported for both boys and girls. As previously stated, gender-scalar measurement invariance was supported.

**Table 3:** Goodness-of-fit statistics of measurement invariance for tested models

Model	S-B χ^2^	df	CFI	TLI	RMSEA	SRMR	Model Comparison	ΔS-Bχ^2^ (Δdf)	Δ CFI
**Measurement invariance across genders**									
Model 1: the one-factor model for boys (*n* = 935)	102.75	35	0.979	0.973	0.045	0.026	-	-	-
Model 2: the one-factor model for girls (*n* = 959)	90.90	35	0.986	0.982	0.041	0.021	-	-	-
Model 3: configural invariance	194.22	70	0.983	0.978	0.043	0.023	-	-	-
Model 4: metric invariance	210.05	79	0.982	0.979	0.042	0.026	*4 vs. 3*	15.84 (9)	− 0.001
Model 5: scalar invariance	230.52	88	0.980	0.980	0.041	0.027	5 vs. 4	20.46 (9)	− 0.002
**Measurement invariance across grades**									
Model 6: the one-factor model for the 3rd graders (*n* = 371)	65.19	35	0.977	0.971	0.048	0.032	-	-	-
Model 7: the one-factor model l for the 4th graders (*n* = 557)	67.64	35	0.984	0.979	0.041	0.026	-	-	-
Model 8: the one-factor model for the 5th graders (*n* = 451)	73.50	35	0.979	0.973	0.049	0.028	-	-	-
Model 9: the one-factor model for the 6th graders (*n* = 515)	68.69	35	0.985	0.980	0.043	0.024	-	-	-
Model 10: configural invariance	275.08	140	0.982	0.976	0.045	0.027	-	-	-
Model 11: metric invariance	323.08	167	0.979	0.977	0.044	0.042	11 vs. 10	47.98 (27)	− 0.003
Model 12: scalar invariance	374.45	194	0.975	0.977	0.044	0.044	12 vs. 11	51.37 (27)	− 0.004

*Note*: Satorra-Bentler scaled ***χ***^***2***^: S-B ***χ***^***2***^; vs.: versus

#### Measurement invariance across grades

All of the one-factor models for different grades (Models 6, 7, 8, and 9) were supported, which showed that the MI could be used across grades. The configural invariance model (Model 10) was also supported, showing that the structure of the AGS was similar between the different grade groups. In the metric invariance model (Model 11), the results showed that despite S-B *χ*^*2*^ significantly increasing (*p* = .003), the ΔCFI between Models 10 and 11 decreased less than the criteria (0.003 < 0.005), supporting metric invariance across grades. In the scalar invariance model (Model 12), except for S-B *χ*^*2*^ significantly increasing (*p* = .003), the change in CFI (0.004) was smaller than.005, indicating scalar invariance across grades. Thus, the scalar MI of the AGS across grades was also supported.

### Structural regression model

**Fig. 4:**
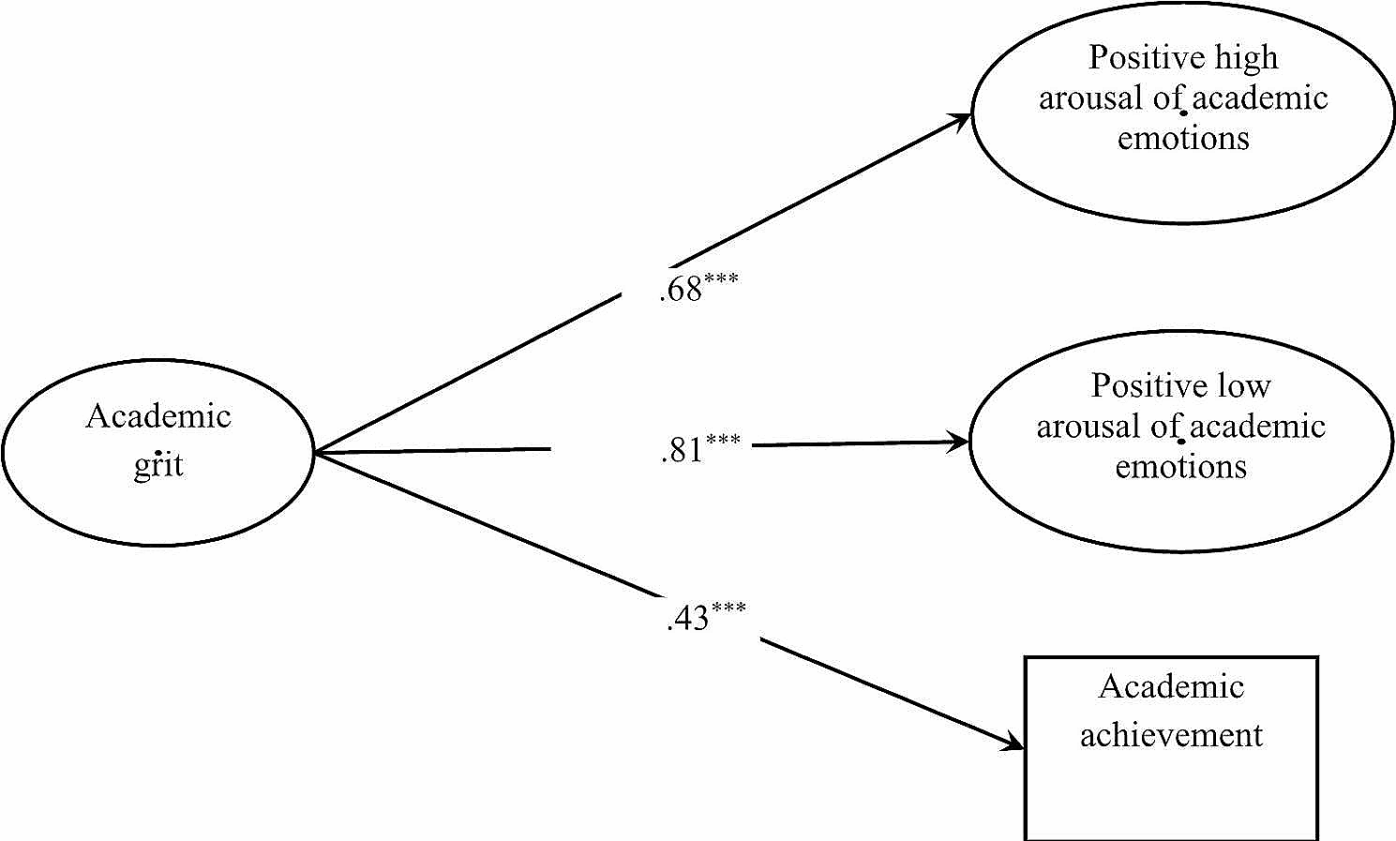
Standardized path coefficients for the structural regression model. *Note*: ^***^: *p* < .001

The one-factor model of the AGS was also used to examine its criteria-related validity. The structural regression model fit the data well: S-B *χ*^2^ = 631.51, *df* = 146, CFI = 0.967, TLI = 0.961, RMSEA = 0.033, and SRMR = 0.045. As shown in Fig. 4, academic grit was positively associated with positive high arousal of academic emotions (*β* = 0.68, *p* < .001), positive low arousal of academic emotions (*β* = 0.81, *p* < .001), and academic achievement (*β* = 0.43, *p* < .001), explaining 46.70%, 65.50%, and 18.10% of the variance in the three variables, respectively. These results indicated that the AGS possessed good predictive validity.

## Discussion

The AGS offers a novel perspective on grit’s role in an academic setting, but it is rarely applied to a new sample or in a new context. This research examined its factor structure and measurement invariance in another culture and a younger sample: Chinese early adolescents. Our primary goal was to overcome some existing issues in AGS regarding its factor structure and measurement invariance and promote its application. On the one hand, the one-factor and three-factor models of AGS were supported by CFA. The bifactor model further showed that the AGS was predominantly explained by a general factor and thus supported a one-factor model of AGS, which is consistent with the findings of Clark and Malecki’s [1] study. The one-factor parallel model of AGS showed good fit indices, whereas the three-factor parallel model of AGS had poor fit indices. It indicates that the sum score of AGS is meaningful while its factor scores are meaningless. On the other hand, MI testing indicated that the AGS’s one-factor model had scalar MI across genders and grades. In short, the current study supports a one-factor structure of the AGS in Chinese early adolescents, as well as the MI in genders and 3rd–6th grader groups. It warrants AGS’s application when measuring Chinese early adolescents’ academic grit.

### Factor structure of the AGS

Prior research lacks a full inspection of AGS’s factor structure as well as its scoring methods. Combating a bifactor model with a parallel model approach, this study is the first attempt to thoroughly examine the factor structure of the AGS among Chinese early adolescents. It makes clear the contribution of AGS’s general factor and three content factors (i.e., determination, resilience, and focus) and ascertains AGS’s scoring methods among Chinese middle- and upper-grade primary school students.

Compared with the CFA, the bifactor model clearly evaluates the extent of homogeneity and heterogeneity of AGS concurrently using several assessment indices. Traditional CFA supported both AGS’s original theoretical model (i.e., the three-factor model of AGS) and the EFA model (i.e., the one-factor model of AGS). It is hard to distinguish the differences between the two models based on CFA. In contrast, the bifactor model more clearly and accurately presented the variance that is explained by the two models. To be specific, the findings indicated that general academic grit was responsible for a large percentage of the AGS’s common variation (general academic grit, 92.0%), item common variation, and item correlations (average *I-ECV* = 0.94, *PUC* = 0.71). These findings remind us that general academic grit may refer to the ability to self-regulate within the academic context. By coordinating the relationship between determination, resilience, and focus, it might urge people to change their thoughts, feelings, and actions to continuously strive to accomplish their academic goals, even when they’re in substantially stressful or unfavorable conditions [1, 6, 38].. Relatively, after the general factor was controlled, three domain-specific factors only explained the small ratio of common variance of the AGS (only 8%), implying that the heterogeneity of the three traits in the AGS is tiny and can be negligible. This means that although the definition of academic girt incorporates three concrete aspects (i.e., determination, resilience, and focus), the homogeneity of the three aspects far outweighs the corresponding heterogeneity, which further supports Clark and Malecki’s [1] univocal model. Going a step further, whether we call it determination, resilience, or focus, it is clear that an individual’s inner strength reveals a common and key ability to a considerable extent: self-regulation. Thus, we labeled their common component (i.e., the general academic grit) as self-regulatory resources.

The parallel model of AGS supports the idea that we can use AGS by summing/averaging all the items. Specifically, the one-factor parallel model of AGS showed good fit indices, whereas the three-factor parallel model of AGS showed poor fit indices. That is, all the items/questions could equally reflect the skill/characteristic of academic grit, while items/questions belonging to specific factors could not equally reflect certain targeted aspects of academic grit: determination, resilience, and focus. Furthermore, when using the measure of AGS, AGS’ sum score could clearly reflect an individual’s level of academic girt, but its factor score cannot well reflect three aspects of academic girt: determination, resilience, and focus. Although the finding fits with researchers’ traditional ideas (i.e., directly use a scale by summing/averaging all the items) [10], the present study provides accurate and sufficient evidence to support AGS’s total score, which to some extent avoids unnecessary biases.

In total, this study thoroughly resolves Clark and Malecki’s [1] contradiction between the theoretical model (i.e., the one-factor model) and the data-based model (i.e., the three-factor model) of the AGS. It gives more clear and robust evidence to support the AGS as an essentially one-factor construct in an Eastern Asian society of early adolescents. Furthermore, we provide more complete evidence to justify reporting AGS as a total score.

### Measurement invariance

Mean differences in academic grit across groups (e.g., boys and girls and 6th–8th graders) were preliminary reported [1], yet measurement invariance—the precondition for a mean comparison—was not evidenced. Due to the developmental levels of psychological and brain structures [17–19], early adolescents in different groups (e.g., genders and grades) may have inconsistent understandings of the contents of the scale items, which may lead to erroneous interpretation of mean differences to a large extent as mean scores mix the group-bias measurement error. This study examined and established the gender and grade scalar MI of the AGS, warranting meaningful and valid mean comparisons among boys and girls as well as 3rd–6th grades. With strong scalar invariance, the gender and grade effects can be truly reflected but not confounded by group-biased measurement issues [13, 14]. Going a step further, with MI, it may be possible to find potential differences in academic grit across genders and grades and ensure the effectiveness of educational interventions. In summary, the AGS can be utilized to make robust and meaningful comparisons and valid conclusions across genders and grades at the observed level.

### Criteria-related validity

This study also demonstrated sufficient criteria-related validity in early adolescents. Academic grit was discovered to be a good predictor of academic achievement and positive high and low arousal of academic emotions. In line with the extant literature [20–24], this result also supports grit playing a critical role in early adolescent academic lives, especially related to affective experiences and behavioral performance. In the Chinese context of high academic pressure and intense academic competition [8, 9], a higher level of academic grit may be helpful for Chinese early adolescents to maintain good academic emotions and facilitate good academic performance. Given the link between grit and well-being [23], the effect of academic grit on positive academic emotions and academic achievement may be the key maintenance factor for early adolescent well-being, whether in physiological or psychological aspects. Besides, the present study also suggests that both domain-general grit (i.e., general grit) and domain-specific grit (i.e., academic grit) have positive effects on early adolescents’ academic and psychological functioning.

## Implications and limitations

Overall, based on the bifactor model and the parallel model, this study warrants the application of AGS’s one-factor structure and its scoring method. With the MI among genders and grades, there is a clear reason for future research to make appropriate comparisons between groups (i.e., genders and grades). There are several important implications for researchers and educators. For researchers, whether in Western or Eastern societies, the AGS is recommended as a unitary model in a SEM context. Also, they can directly compare the gender and grade differences with the mean values among Chinese early adolescents. For educators, it is meaningful to report the total score when they use the AGS to assess adolescent academic grit.

Several limitations must be taken into account. First, this study only looked at how the AGS was used among early adolescents, specifically those in the middle and upper grades of elementary school. Future work should look into the AGS’s applicability to other age groups, such as middle and late adolescents and even college students, as well as children in lower grade levels and preschool. Second, culture and language have been considered vital sources of measurement error [39]. Future researchers should create cross-cultural MI to confirm the coherence of the structure of academic grit between Eastern and Western nations. Similarly, to better detect the developmental effect of academic grit, the examination of test-retest reliability and longitudinal MI is also recommended. Furthermore, the criteria-related variables in this study were all measured using a self-reported cross-sectional method, which may be affected by common method bias. Future research should adopt other forms of reporting and longitudinal analysis to fully reflect the predictive validity of the AGS.

### Electronic supplementary material

Below is the link to the electronic supplementary material.

Supplementary Material 1

## Data Availability

The datasets generated during and/or analysed during the current study are available from the first corresponding author on reasonable request.

## Footnotes

1 Given that academic grit is a crucial characteristic or skill associated with academic potential and performance, selecting Chinese early adolescents attending school rather than those who have dropped out is more appropriate as our research focus. Additionally, during this period, the majority of Chinese early adolescents are enrolled in the middle or upper grades of primary school. Consequently, our research is centered around Chinese middle- and upper-grade primary school students.

2 A parallel model is derived from a corresponding factor model. For example, AGS’s three-factor parallel model is derived from AGS’s three-factor model. Similarly, AGS’s one-factor parallel model is derived from AGS’s one-factor model. In the factor model, the error variance and loadings remain unconstrained. In contrast, the parallel model sets factor loadings equal for all items and constrains the error variance of items to be unique across factors but equal within the same factor [10].

3 MLR is a robust estimation method that is less sensitive to violations of normality assumptions and is suitable for data that may not be perfectly normally distributed [30, 31].

4 A second-order model of AGS was also tested due to the high correlations between the three factors of AGS’s three-factor model. However, we consider the theoretical basis, the results of previous research by Clark and Malecki’s (2019), and the research questions and decide not to present the results here. The results and concrete explanation can be seen in the supplemental material 1.
