# Supplementary material for: Academic grit scale for Chinese middle- and upper-grade primary school students: testing its factor structure and measurement invariance
Source: BMC Psychol. 2024 Mar 14;12:149. doi: 10.1186/s40359-024-01622-y (PMC10941363; doi:10.1186/s40359-024-01622-y)
Supplement: Supplementary file 1 — Supplementary Material 1 [file 40359_2024_1622_MOESM1_ESM.docx]

**Appendix**

Academic Grit Scale (Clark & Malecki, 2019)

Chinese version follows English version.

Items 1, 5, 9, and 10 are about determination; Items 2, 4, 6, and 8 are about resilience; and Items 3 and 7 are about focus.

1. I push myself to do my personal best in school.

1. 在学业上，我要求自己努力做到最好。

2. I work toward my academic goals no matter how long they take to reach.

2.无论多么艰难，我都会一直朝着自己的学习目标努力。

3. Even when I could do something more fun, I give schoolwork my best effort.

3.即使有其它更有趣的事情，我仍然会先尽自己最大的努力把学业任务完成好。

4. I complete my schoolwork no matter how difficult it is.

4.无论功课或作业有多难，我都会努力完成它。

5. I am determined to give my best effort in schoolwork.

5.我决定在学业上尽自己最大的努力。

6. Once I set a goal in school, I try to overcome any challenges that arise.

6.一旦确立了学习目标，我会努力克服遇到的各种困难。

7. I am able to balance working hard in school with my other hobbies and interests.

7.我能够处理好刻苦学习与其它兴趣爱好之间的关系。

8. Even if I am struggling in school, I keep trying my best.

8.即使学业辛苦，我也坚持做到最好。

9. When it comes to completing work in school, I always try my hardest.

9.在完成课业方面，我总是尽最大努力。

10. In school, I work hard to achieve challenging goals.

10.在学业上，我努力去完成富有挑战性的目标
